# Design and numerical simulation of CuBi_2_O_4_ solar cells with graphene quantum dots as hole transport layer under ideal and non-ideal conditions

**DOI:** 10.1038/s41598-024-83700-0

**Published:** 2025-01-02

**Authors:** Muhammad Panachikkool, E. T. Aparna, Perumal Asaithambi, T. Pandiyarajan

**Affiliations:** 1 Department of Sciences, Indian Institute of Information Technology Design and Manufacturing Kurnool, Kurnool, Andhra Pradesh 518008 India; 2 Faculty of Civil and Environmental Engineering, Jimma Institute of Technology, Jimma University, Po Box – 378, Jimma, Ethiopia

**Keywords:** Solar cell, SCAPS 1D simulator, CuBi_2_O_4_, GQDs, Energy science and technology, Renewable energy, Solar energy, Photovoltaics, Solar cells

## Abstract

The simulation of ideal and non-ideal conditions using the SCAPS-1D simulator for novel structure Ag/FTO/CuBi_2_O_4_/GQD/Au was done for the first time. The recombination of charge carriers in CuBi_2_O_4_ is an inherent problem due to very low hole mobility and polaron transport in the valence band. The in-depth analysis of the simulation result revealed that Graphene Quantum Dots (GQDs) can act as an appropriate hole transport layer (HTL) and can enhance hole transportation. The simulation was done under ideal and nonideal conditions. The non-ideal conditions include parasitic resistances, reflection losses, radiative, and Auger recombination whereas the ideal condition was studied without the inclusion of any losses. Under ideal conditions, the cell Ag/FTO/CuBi_2_O_4_/GQD/Au exhibited a photovoltaic (PV) parameter such as open circuit voltage (V_oc_), short circuit current (J_sc_), fill factor (FF), photo conversion efficiency (PCE) are 1.39 V, 25.898 mA/cm^2^, 90.92%, and 32.79%, respectively. The effect of various cell parameters such as the thickness of the absorber layer, HTL layer, and FTO, acceptor and defect density, the bandgap of the absorber and HTL layer, series and shunt resistance, back and front contact materials, radiation and Auger recombination of the absorber layer, reflection losses on the efficiency of the proposed cell is analysed. The drastic reduction in all PV parameters was observed under non-ideal conditions and the PV parameters are V_oc_ (1.22 V), J_sc_ (2.904 mA/cm^2^), FF (86.3), and PCE of 3.06%. The charge kinetics such as impedance, conductivity, and capacitance plots, and possible reasons for reductions in PV parameters are discussed in detail.

## Introduction

The climatic changes begin with non-renewable energy usage and carbon emission^1^ and adverse effects of climatic changes are hitting hard daily on the Earth^2^. According to the International Energy Agency (IEA) Report, in 2023, there is a huge increment in carbon emissions from 410 million tonnes to 37.4 billion tonnes and the major portion (65%) of this originated from coal^3^. According to the United Nations, Paris Agreement (2015), the net carbon emissions are set to be zero by 2050, and the global temperature increment is to hold at 1.5 °C^4^. To achieve these, renewable energy sources best alternative to fossil fuels and cut down carbon emissions. The sun is the main source of energy for Earth, and there is a tremendous amount of energy, which is equal to 1.73 × 10^14^ kW which is 10,000 times more than actual energy demands^5^. The photo voltaic cell is used to convert solar energy into electrical energy and could be useful to overcome the issues of carbon emission and global warming driven by fossil fuels^6^. Silicon-based SCs contribute the major share in the solar cell (SCs) industries^7^; however, the maximum efficiency of these Silicon-based SCs is about 27.1%^8^ on the lab’s scale and 23% on the module scale^9^. Nevertheless, the process of purification of silicon wafers for SCs is tedious, material cost is also high, and the theoretical maximum PCE is also limited. To tackle these issues, various new-generation SCs are introduced, which have the potential to achieve high efficiency, an easy process that includes second^10^, third^11^, and fourth-generation SCs^12^. Second-generation SCs mainly consist of thin film-based SCs like CdTe, CIGS, and GaAs SCs which exhibit good efficiency 22.6% (CdTe), 23.6% (CIGS)^8^, still the scarcity and the cost of materials are high^13^.

Copper bismuth oxide (CuBi_2_O_4_) is a promising material for SCs due to its high absorption coefficient (> 10^4^ cm^-1^)^14^, and tuneable band gap (1.35–1.80 eV)^15^. However, low hole mobility and small diffusion length (~ 10–45 nm)^16^ are the main hurdles to producing highly efficient SCs. So far, there are no experimental studies on CuBi_2_O_4_-based SCs and a few simulation studies using SCAPS-1D simulators in ideal conditions^17–22^ and non-ideal conditions^23^. Adnan et al.^17^ reported firstly the simulations studies using CuBi_2_O_4_ as an absorber layer with a CdS Buffer layer, in a cell configuration of Al/SnO_2_:F(FTO)/CdS/CuBi_2_O_4_/Ni and achieved a PCE of 26% with PV parameters of V_oc_ (0.97 V), J_sc_ (31.61 mA/cm^2^), and FF of 84.55%. Yswanth and co-workers^18^ simulated the cell structure with different metal sulfides (CdS, ZnS, SnS_2_, and WS_2_) as buffer layers for a simple structure of FTO/Metal sulfides/CuBi_2_O_4_/Au. The maximum PCE was achieved for WS_2_ (22.84%) and other sulfides such as CdS (21.30%), SnS_2_ (20.94%), and ZnS (22.30%) were also obtained. Manjunath et al*.*^19^ replaced the metal sulfide buffer layers with ABO_3_ perovskite materials such as SrTiO_3_, BaTiO_3_, SrSnO_3_, and BaSnO_3_ with a cell configuration of glass/ITO/ABO_3_ Buffer/CuBi_2_O_4_/Au structure and achieved a PCE of 22.19% for SrSnO_3_ buffer layer. Kushal et al*.*^20^ simulated the TiO_2_ buffer layer with a cell configuration of Al/ITO/TiO_2_/CuBi_2_O_4_/Mo and reached and high PCE of 31.21%. Santhosh et al.^21^ used multiple metal oxide transport layers including WO_3_, SnO_2_, ZnO, and TiO_2_, for a configuration of glass/ITO/Metal oxide Buffer/CuBi_2_O_4_/Au and showed theoretical efficiency of 26.18% (WO_3_), 25.93% (SnO_2_), 26.13% (ZnO), and 26.03% (TiO_2_). Adnan et al.^22^ simulated by keeping the CdS buffer layer, with different HTLs such as Cu_2_O, CuI, NiO_x_, MoO_3_, and Sb_2_S_3_ with a configuration of Al/FTO/CdS/CuBi_2_O_4_/HTL/Mo. The maximum achieved PCE of 29.2% is obtained for Cu_2_O as HTL. However, all these conditions are done under ideal conditions, and actual cell performance would highly depend on non-ideal conditions.

Seyyed et al*.*^24^ first reported the performance of SCs on non-ideal conditions with the cell structure of FTO/TiO_2_/CH_3_NH_3_PbI_3_/spiro-OMeTAD/Au. Interestingly, it was observed that a drastic reduction of PCE from 19.26% to 8.40% for ideal and non-ideal conditions, respectively. This reduction is obtained by considering nonideal factors such as parasitic resistance, reflection losses, and recombination losses. Further, optimizing the spiro-OMeTAD, champion cells achieved a PCE of 12.81% which is very close to the experimental value of 12.71%. Shahariar et al.^25^ also have done similar non-ideal conditions in the cell structure of FTO/TiO_2_/Sb_2_Se_3_/spiro-OMeTAD/Au in ideal and non-ideal conditions, the PCE is a downfall from 25% to 8.40%. Non-ideal conditions are required to validate the experimental performance of SCs. By adopting this approach, we previously reported^23^ the decrement in PCE from 31.23% to 0.46% for a cell configuration of Mo/CuBi_2_O_4_/TiO_2_/ITO/Al from ideal to non-ideal condition. This low efficiency is due to the high recombination factor, and low hole mobility due to polaron transportation^26^. To tackle these issues, new materials are needed as hole transport layer (HTL) to extract the charge carriers rapidly before the recombination occurs. Efforts have been made to simulate real conditions by comparing simulations with experimental results. Hajjiah and his team were analyzed the thin-film SCs using the Sah–Noyce–Shockley recombination model to study their current-voltage characteristics^27^.

The GQDs are the zero-dimensional^28^, carbon allotrope with sp^2^ hybridized carbon atoms with a hexagonal structure, size-dependent band gap, and luminescence properties. The GQDs have been used in a wide range of applications such as photovoltaics, photocatalysis, LEDs, and laser applications^29^. The GQDs could be effectively used as HTL^30^, electron transport layer^31^, active layer^32^, and counter electrode^33^ in SCs. Further, GQDs are used in SCs as dopants, decoration, and composite with PEDOT: PSS, and perovskite materials^34^. The GQDs not only enhance the PCE but also enhance the stability^35^, and durability in flexible SCs, and act as a protective layer in perovskite SCs^36^. To the best of our knowledge, no report in simulation and experimental studies using GQDs as HTL in CuBi_2_O_4_-based SCs.

Owing to the importance of GQDs and CuBi_2_O_4_ in PV industries, in this report, we have performed simulation studies on Ag/FTO/CuBi_2_O_4_/GQD/Au cell structures under both ideal and non-ideal conditions. We have evaluated the effect of various cell parameters such as the thickness of the absorber layer, HTL layer, and FTO, acceptor and defect density, the bandgap of the absorber and HTL layer, series and shunt resistance, back and front contact materials, radiation and Auger recombination of the absorber layer, reflection losses on the efficiency of the proposed cell are analyzed in detail.

## Device architecture and simulations

The solar cell capacitance simulator for one dimension (SCAPS-1D) by one-dimensional solar cell simulation program was designed and developed at the Department of Electronics and Information Systems (ELIS) of the University of Gent, Belgium. In the present manuscript, we have used SCAPS simulation software with version 3.3.10 (https://scaps.elis.ugent.be/SCAPSinstallatie.html).

The SCAPS 1D software is based on the semiconductor equations in steady-state conditions^19^. The relationship between electric field and space charge density can determined by the Poissons equation (Eq. 1) and the information on charge carrier transport can be obtained by solving the continuity equation of electrons and holes (Eqs. 2 and 3), drift and diffusion of electrons (Eq. 4) and hole equations (Eq. 5). By inserting appropriate material properties for each material in the layers, the software could obtain the PV parameters by solving these equations^19^

1 $$\frac{dE}{dx}=-\frac{{d}^{2}\psi }{d{x}^{2}}=\frac{q}{\varepsilon }\left[p\left(x\right)-n\left(x\right)+{N}_{D}^{+}\left(x\right)-{N}_{A}^{-}+{p}_{t}\left(x\right)-{n}_{t}\left(x\right)\right]$$

where, *Ψ*: electrostatic potential, *q*: charge of the electron, *ε*: Dielectric constant of the material, *p*: hole concentration, *n*: electron concentration, *N*_*A*_^*-*^: density of ionized acceptors, *N*_*D*_^+^: density of ionized donors, *n*_*t*_: trapped electrons, *p*_*t*_: trapped holes, *x*: position coordinates^19^

2 $$\frac{d{n}_{}}{dt}={G}_{n}-\frac{{n}_{}-{n}_{}^{0}}{{\tau }_{n}}+{n}_{}{\mu }_{n}\frac{dE}{dx}+{\mu }_{n}E\frac{d{n}_{}}{dx}+{D}_{n}\frac{{d}^{2}{n}_{}}{d{x}^{2}}$$

3 $$\frac{d{P}_{}}{dt}={G}_{p}-\frac{{p}_{}-{P}_{}^{0}}{{\tau }_{p}}+{p}_{}{\mu }_{p}\frac{dE}{dx}+{\mu }_{p}E\frac{d{P}_{}}{dx}+{D}_{p}\frac{{d}^{2}{P}_{}}{d{x}^{2}}$$

were*, G*_*n*:_ electron generation rate *G*_*p*_: hole generation rate *n*: electron in the *p* region, *p*: hole concentration *n* region, *n*^0^: equilibrium electron concentration in *p* region, *p*^0^: equilibrium hole concentration in *n* region, *τ*_*n*_: electron lifetime, *τ*_*p*_: hole lifetime, *µ*_*p*_: hole mobility, *µ*_*n*_: electron mobility, *E:* electric field, *D*_*n*_: electron diffusion coefficient, *D*_*p*_: hole diffusion coefficient^19^

4 $${J}_{n}\left(x\right)=qn{\mu }_{n}E+q{D}_{n}\frac{dn}{dx}= n{\mu }_{n}\frac{d{E}_{Fn}}{dx}$$

5 $${J}_{p}\left(x\right)=qp{\mu }_{p}E+q{D}_{p}\frac{dp}{dx}= p{\mu }_{p}\frac{d{E}_{Fp}}{dx}$$

E_fn_: Quasi-fermi levels of electrons, E_fp_: Quasi-Fermi level of holes

**Fig. 1:**
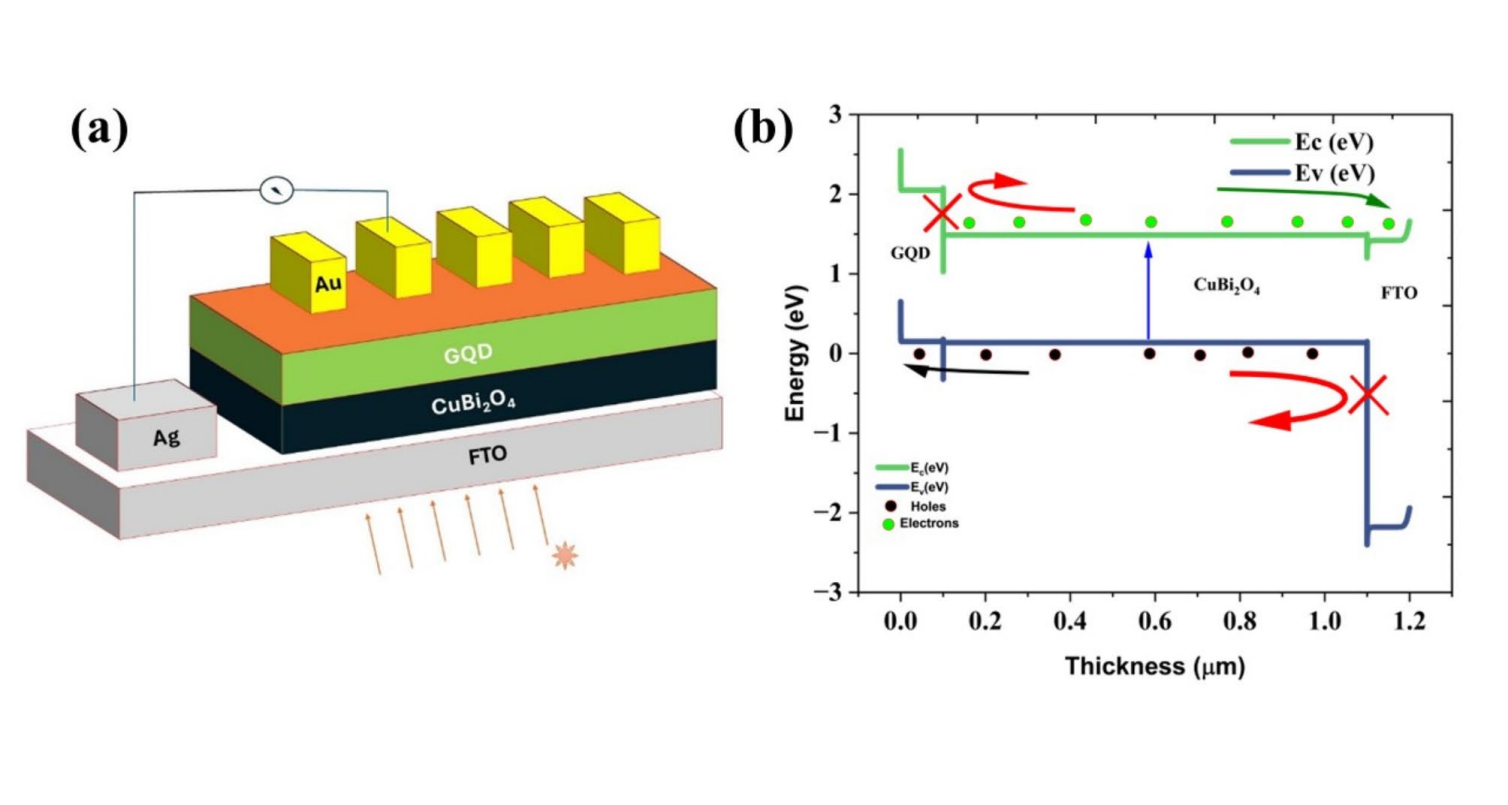
(**a**) The schematic diagram of the proposed cell structure in the configuration of Ag/FTO/CuBi_2_O_4_/GQD/Au, where FTO, CuBi_2_O_4_, GQD, Ag, and Au are transparent conducting electrodes, the absorber layer, HTL, front contact, and back contact, respectively. (**b**) band structure the black dots represent holes and the green dots represent electrons. The blue line and green lines represent the valence and conduction band energies, respectively. The black arrows show the flow of holes, the green arrow shows the flow of electrons, and the red arrows represent the blockage of carriers.

The proposed cell structure with the configuration of Ag/FTO/CuBi_2_O_4_/GQD/Au, where Ag and Au are the front and back contact, FTO is the window layer, CuBi_2_O_4_ is the absorber layer, and GQD acts as HTL depicted in Fig. 1a. The simulation is done under the conditions of AM 1.5 G illuminations with a power density of 100 mW/cm^2^ and a temperature of 300 K. The band diagram of the final cell is depicted in the Figure, and it shows that the bands match perfectly. Here, The GQDs can act as a good HTL, the valence band of the absorber layer and the HTL are very close, which reveals that the GQD can act as a good HTL. In addition to this, the difference in the conduction band of GQD and CuBi_2_O_4_ is high, which block the electron on the other side (Fig. 1b).

## Results and discussion

### Ideal conditions

In ideal conditions, the variation of parameters such as the band gap, thickness, and acceptor densities, for both the absorber layer and HTL, and the work function of the front and back contact layer. The work functions of the front contact (Ag) and back contact (Au) layers are set as 4.26 eV and 5.2 eV. The material parameters for the ideal condition are listed in Table 1. The CuBi_2_O_4_/GQD interface parameters are also listed in Table 2.

**Table 1:** The material parameters for ideal conditions.

Parameters	FTO^17^	CuBi_2_O_4_ (Absorber Layer)^17–22^	GQD (HTL)
Thickness (nm)	50	Variable	variable
Band gap (eV)	3.6	1.35- 1.80	2.0–3.0^37^
Electron Affinity (eV)	4.0	3.72	2.650^38^
Dielectric permittivity	9	34	700^39^
CB effective DOS (cm^−3^)	2.2 × 10^18^	1.2 × 10^19^	3 × 10^19^
VB effective DOS (cm^−3^)	1.8 × 10^18^	5.0 × 10^19^	3 × 10^19^
Electron mobility (cm^2^ V^−1^ S^−1^)	20	1.1 × 10^–3^	100
Hole mobility (cm^2^ V^−1^ S^−1^)	10	1.2 × 10^–3^	2.45^40^
Donor density, N_D_ (cm^−3^)	1.0 × 10^18^	0	0
Acceptor density, N_A_ (cm^−3^)	0	1.0 × 10^19^	1 × 10^18^

**Table 2:** The Defect parameters used in the CuBi_2_O_4_ /GQD interface.

Parameters (units)	GQD/CuBi_2_O_4_ Interface
Defect type	Neutral
Capture cross section electrons (cm^2^)	1.00 × 10^–19^
Capture cross-section holes (cm^2^)	1.00 × 10^–19^
Energetic distribution	Single
Reference for defect energy level E_t_	Above the highest E_V_
Energy with respect to Reference (eV)	0.600
Total Density	1.00 × 10^10^

#### The effect of layer thickness

**Fig. 2:**
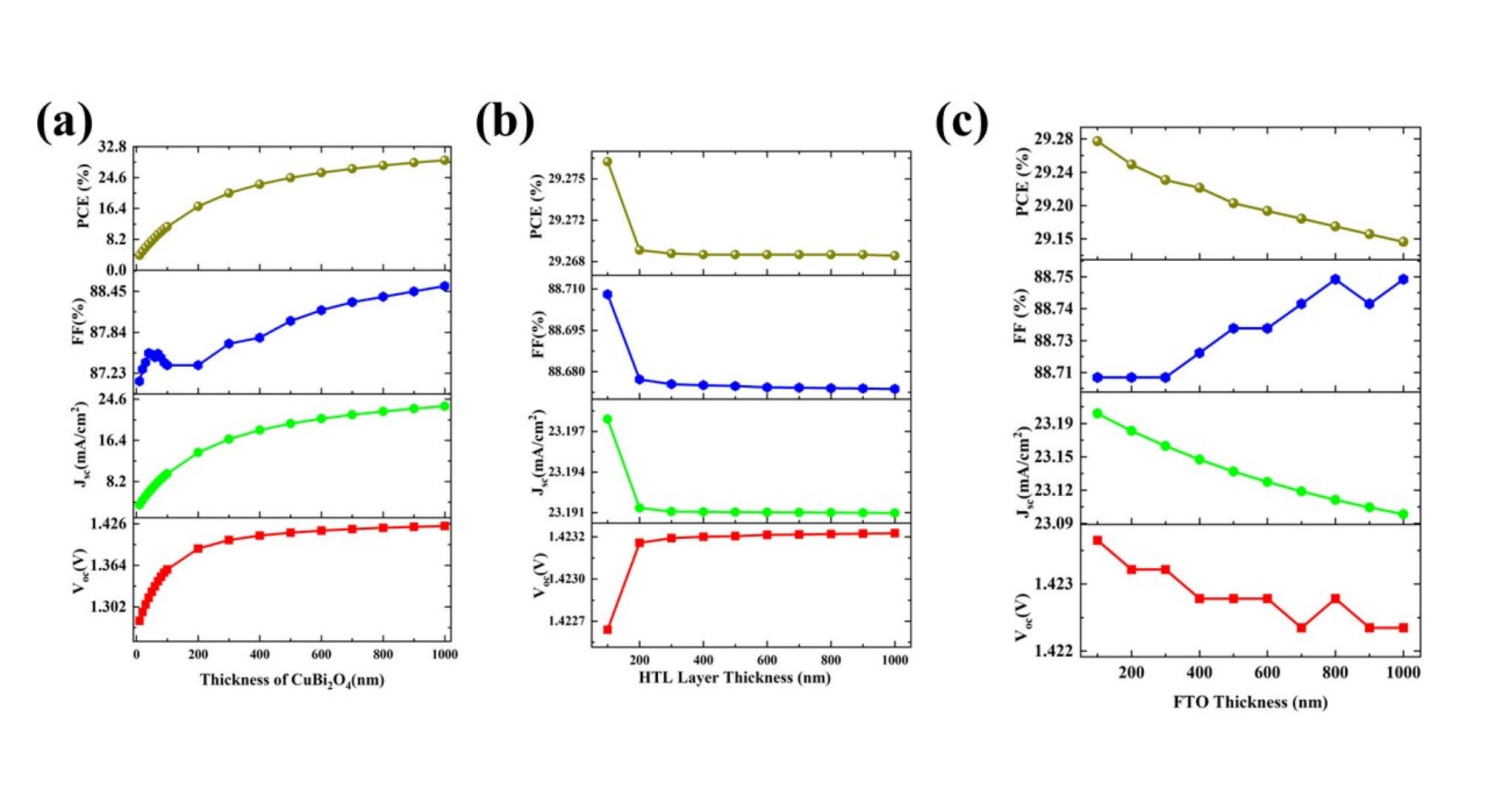
The variation in photovoltaic parameters, including V_oc_, J_sc_, FF, and PCE, under ideal conditions for the configuration Ag/FTO/CuBi_2_O_4_/GQD/Au with respect to: (**a**) varying absorber layer thickness, (**b**) varying HTL layer thickness, and (**c**) varying FTO layer thickness.

The absorber layer thickness is an important parameter for absorbing light photons and generating excitons^41^. Here, the thickness of the absorber layer varied from 10 to 1000 nm. All PV parameters are increased upon the increase of thickness (Fig. 2a), and this is mainly due to an increment in the absorption of more photons and the generation of more excitons^18^. The PCE has increased from 3.98% to 29.22% for 10 nm and 1000 nm thickness, respectively. All other PV parameters such as V_oc_ (1.281 V to 1.422 V), J_sc_ (3.568 mA/cm^2^ to 23.198 mA/cm^2^), and FF (84.11% to 87.35%) also increased.

The thickness of the HTL is varied from 100 to 1000 nm with a fixed absorption layer thickness of 1 μm (Fig. 2b). It is observed that there is a small decrement in all PV parameters such as J_sc_ (23.198 mA/cm^2^ to 23.191 mA/cm^2^), FF (88.71% to 88.68%), and PCE (29.28% to 29.27%) except V_oc_ which increased a slightly (1.422 V to 1.423 V) upon the increase in thickness from 100 to 200 nm. Interestingly, with a further increase of HTL thickness, all the PV parameters are constant up to 1000 nm. This indicates that there is not much influence on thickness in the PV parameters and a similar result was reported in the literature^22^. Due to the light blocking of the absorption layer, HTL will not influence the absorption and current generation. The layer thickness of 100 nm is sufficient for the extraction of holes from the absorber layer and fixed HTL of 100 nm for further optimization of SCs.

The FTO acts as a window layer, transparent conducting electrode in this configuration. The thickness of FTO varies from 100 to 1000 nm and with the increase in the thickness of FTO, there is a slight decrement in the current generation (J_sc_ is from 23.19 mA/cm^2^ to 23.11 mA/cm^2^) which is due to the reduction of light reaching the absorber layer (Fig. 2c). This decrement in current affects the PCE and there is a slight decrease in PCE from 29.28% to 29.17%. The V_oc_ is almost constant (1.4227 V to 1.4225 V), and FF is slightly increased from 88.71% to 88.74%. Considering the PCE reduction and PV parameters, the best thickness for FTO is fixed as 100 nm with PV parameters of V_oc_ (1.423 V), J_sc_ (23.198 mA/cm^2^), FF (88.71%), and a PCE of 29.28%.

#### The effect of acceptor densities

**Fig. 3:**
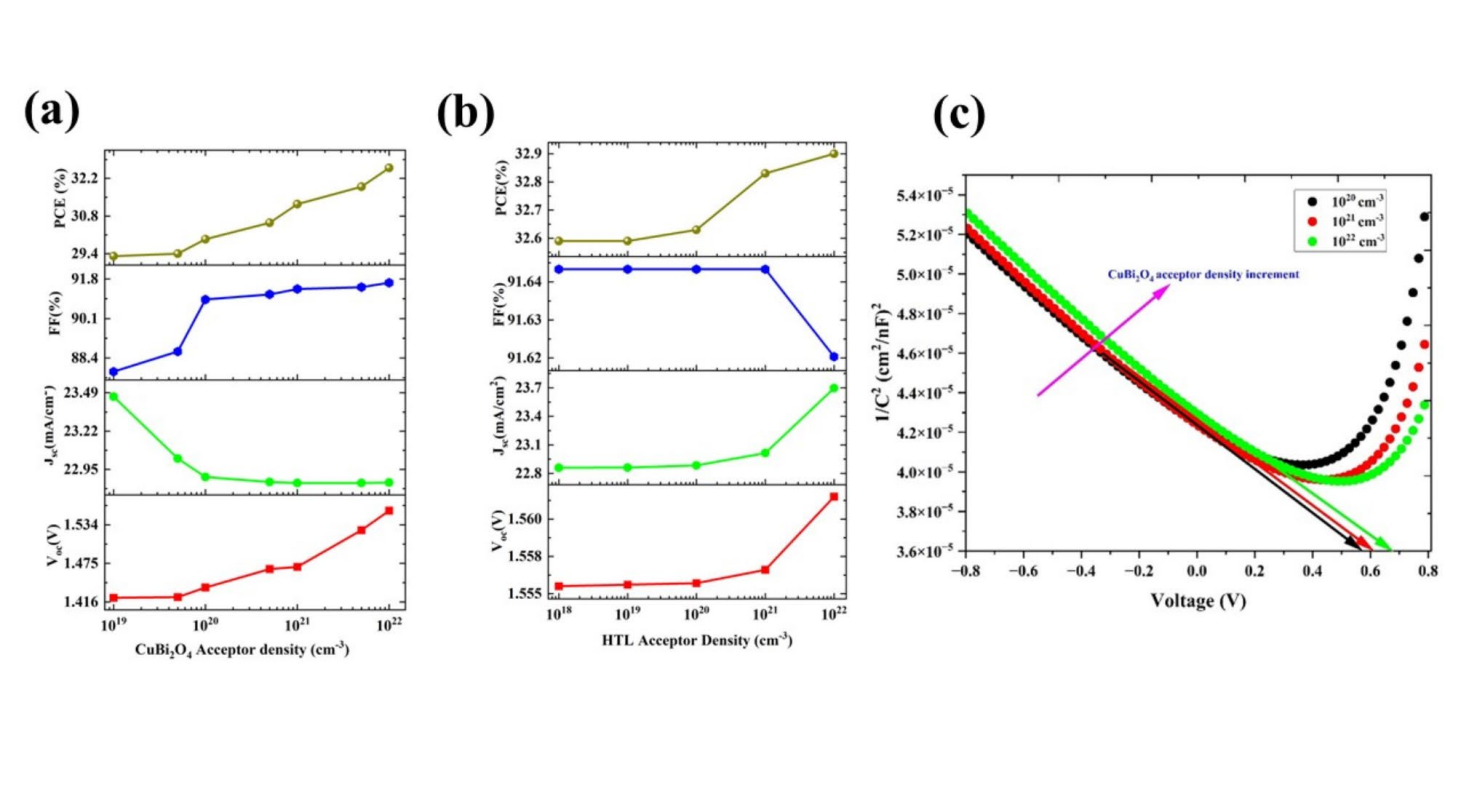
The variation of photovoltaic parameters V_oc_, J_sc_, FF, and PCE with respect to acceptor densities of the (**a**) absorber layer, (**b**) HTL layer, and (**c**) Mott Shotkey plot for absorber layer acceptor densities 10^20^ cm^−3^ (black dotted), 10^21^ cm^−3^ (red dotted), and 10^22^ cm^−3^ (green dotted), for the configuration Ag/FTO/CuBi_2_O_4_/GQD/Au under ideal conditions. The arrows show the built-in potential for each case of acceptor densities.

The CuBi_2_O_4_ is inherently a *p*-type material, and the acceptor density varied from 10^19^ cm^–3^ to 10^22^ cm^−3^, except for current density J_sc_ (23.46 to 22.85 mA/cm^2^), all other PV parameters are increased V_oc_ from 1.422 V to 1.555 V, FF from 87.81% to 91.64 and PCE from 29.31% to 32.59% (Fig. 3a). The current density is decreasing due to the increment of hole concentration which will act as trap centers that will reduce the current and more number carrier concentration will uplift the voltage and smooth passage of carriers would enhance the FF^22^. This increment in voltage may be due to the downward shift fermi level towards the valance band edge, which will increase the built-in potential and hence overall performance. The built-in potential was an important parameter that greatly affected the trap-assisted recombination and can be calculated from the CV analysis by the Mott-Schottky plot and built-in potential could be extracted from the following formula^15^.

6 $$\frac{1}{{C}^{2} }=\frac{2}{{\varepsilon }_{0}\varepsilon {A}^{2}e{N}_{A}}(-\varphi +{\varphi }_{bi}-\frac{kT}{e})$$

where* C* is the capacitance per unit area, ${\varepsilon }\,\,and\,\, \varepsilon_{0 }$ are relative permittivity and permittivity of free space, *e* is charge of the electron, *N*_*A*_ is the acceptor density, *T* is the temperature (set as 300 K), *k* is the Boltzmann constant, and the ${\varphi }_{bi}$ is the built-in potential. Figure 3c clearly shows that the built-in potential increases would contribute to the enhancement in PV parameters via reducing recombination. For the N_A_ is 10^22^ cm^−3^, the built-in potential is about 0.64 V which is reported in the literature^22^. To achieve better efficiency, the optimized acceptor density of the absorber layer is fixed at 10^22^ cm^−3^.

The acceptor density of HTL varies from 10^19^ cm^−3^ to 10^22^ cm^−3^ and all PV parameters except the FF (91.64 to 91.62%) are increased (Fig. 3b). The V_oc_, J_sc_, and PCE are increased from 1.55 V to 1.56 V, 22.85 mA/cm^−2^ to 23.69 mA/cm^2^, 32.59% to 33.90% respectively. The increment in PCE implies that the acceptor density is adequate for the hole extraction from the cell. However, for the superior performance of the cell, the acceptor density of GQD is kept at 10^22^, and the PV parameters V_oc_, J_sc_, FF, and PCE are 1.561 V, 23.69 mA/cm^2^ 91.62%, and 33.90%, respectively.

#### The effect of band gap

**Fig. 4:**
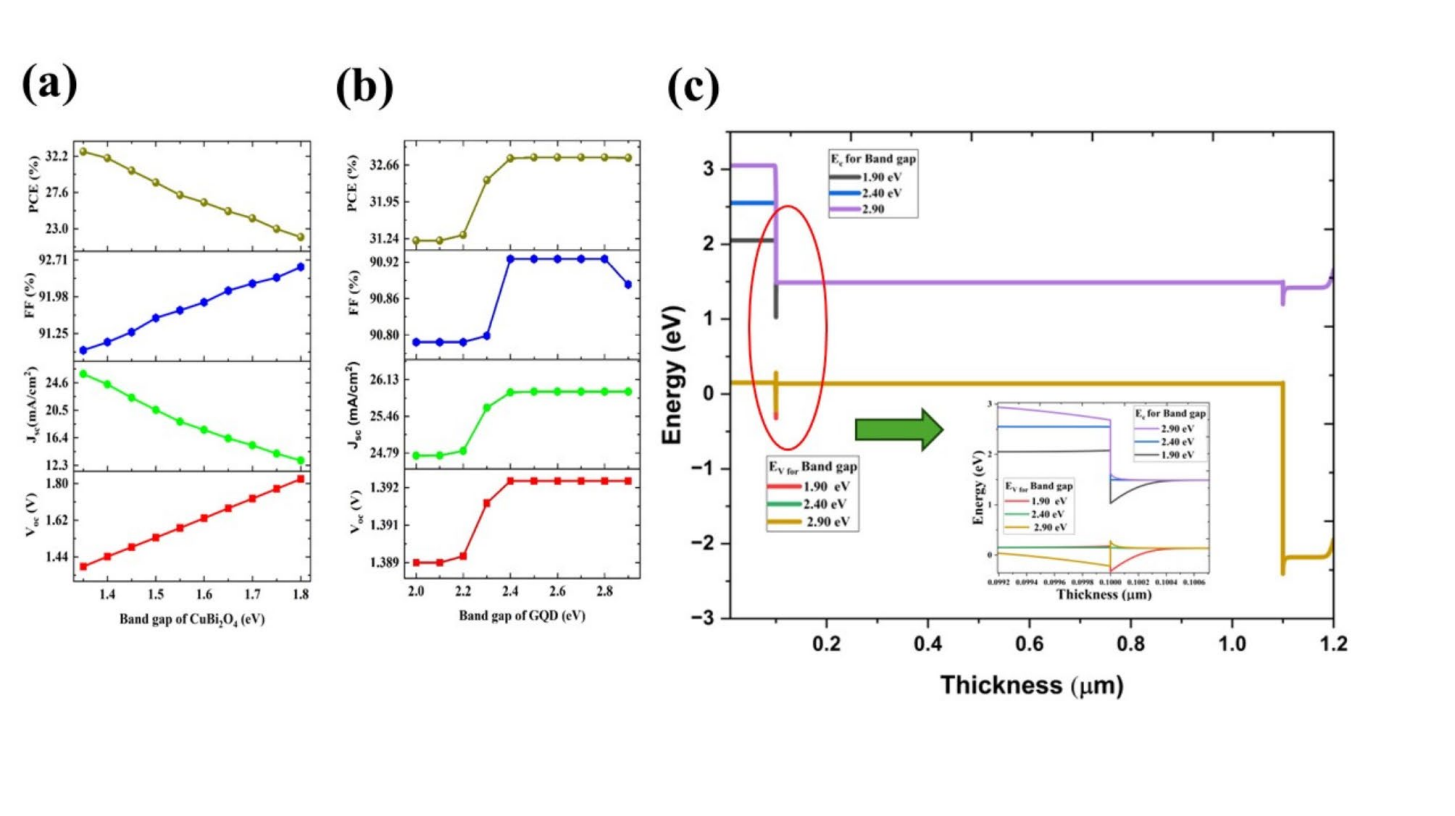
The variation of photovoltaic parameters (V_oc_, J_sc_, FF, and PCE) upon the variation of the band gap of (**a**) CuBi_2_O_4_ (**b**) GQD for the configuration Ag/FTO/CuBi_2_O_4_/GQD/Au under ideal conditions. (**c**) The band alignment diagram for the cell structure of Ag/FTO/CuBi_2_O_4_/GQD/Au with a varying bandgap of GQDs as 1.90 eV, 2.40 eV, and 2.90 eV and the zoomed version of the interface are shown in the inset. The red, green, and yellow represent the valence band energy for the GQD of bandgaps of 1.90 eV, 2.40 eV, and 2.90 eV respectively. Black, blue, and violet are the conduction bands for the bandgaps GQDs of 1.90 eV, 2.40 eV, and 2.90 eV, respectively.

The band gap is one of the crucial criteria for selecting the appropriate material for photovoltaics. Experimental studies are showing that the CuBi_2_O_4_ can be tuned from 1.35 V to 1.75 V^15^. In the present studies, the band gap has varied from 1.35 V to 1.8 V with a step of 0.05 V (Fig. 4a). The increment of the band gap would increase the V_oc_ and FF and there will be decrement in J_sc_. The decrement in current density is due to the reduction of the number of excitons due to the absorption of low-energy photons than the band gap. In short, the band gap is a crucial parameter, which can enhance only current, or voltage based on the variation of decrement or increment. In this case, the highest efficiency is obtained at a low band gap (1.35 eV), due to the high absorption coefficient of the absorber layer will compensate for the voltage decrement by a comparatively high increment in current. The maximum PCE was obtained at a band gap of 1.35 eV with a V_oc_ of 1.392 V, J_sc_ 25.899 mA/cm^2^, and FF of 90.92%.

The GQD is a quantum material that can easily alter the band gap with size, defects, or functional groups^42^. Initially, the band gap has varied from 1.5 eV to 3.5 eV*.* However, the band gap discrepancies with the absorber layer are observed below 1.90 eV and above 3.0 eV. Further analysis, we varied the band gap from the 2.0 eV to 2.90 eV with a step size of 0.10 eV. By increasing the band gap from 2.0 eV to 2.4 eV, the PCE is enhanced from 31.20 to 32.80%, further increasing of bandgap to 2.9 eV, PCE is saturated (Fig. 4b). This result is consistent with the band gap alignment (Fig. 1b), at 2.40 eV valence band perfectly matching with the valence band of the absorber layer.

The increment and reduction of the bandgap would give more barrier between the valence between HTL and the absorber layer (Fig. 4c). The reduction of the band gap below 1.90 V may cause the electron flows between the conduction band of HTL and the absorber layer may cause a further reduction in the current (Fig. 4c inset). Here, there is another observation, which can easily be obtained, the appropriate band gap of GQD, can act as good HTL and also act as electron electron-blocking layer. The 2.4 eV is the appropriate optimized band for the HTL material, and the PV parameters obtained for the cell are 1.3926 V (V_oc_), 25.89 mA/cm^2^ (J_sc_), 90.92% (FF), and 32.80% (PCE).

#### The Effect work function of the back contact layer

**Fig. 5:**
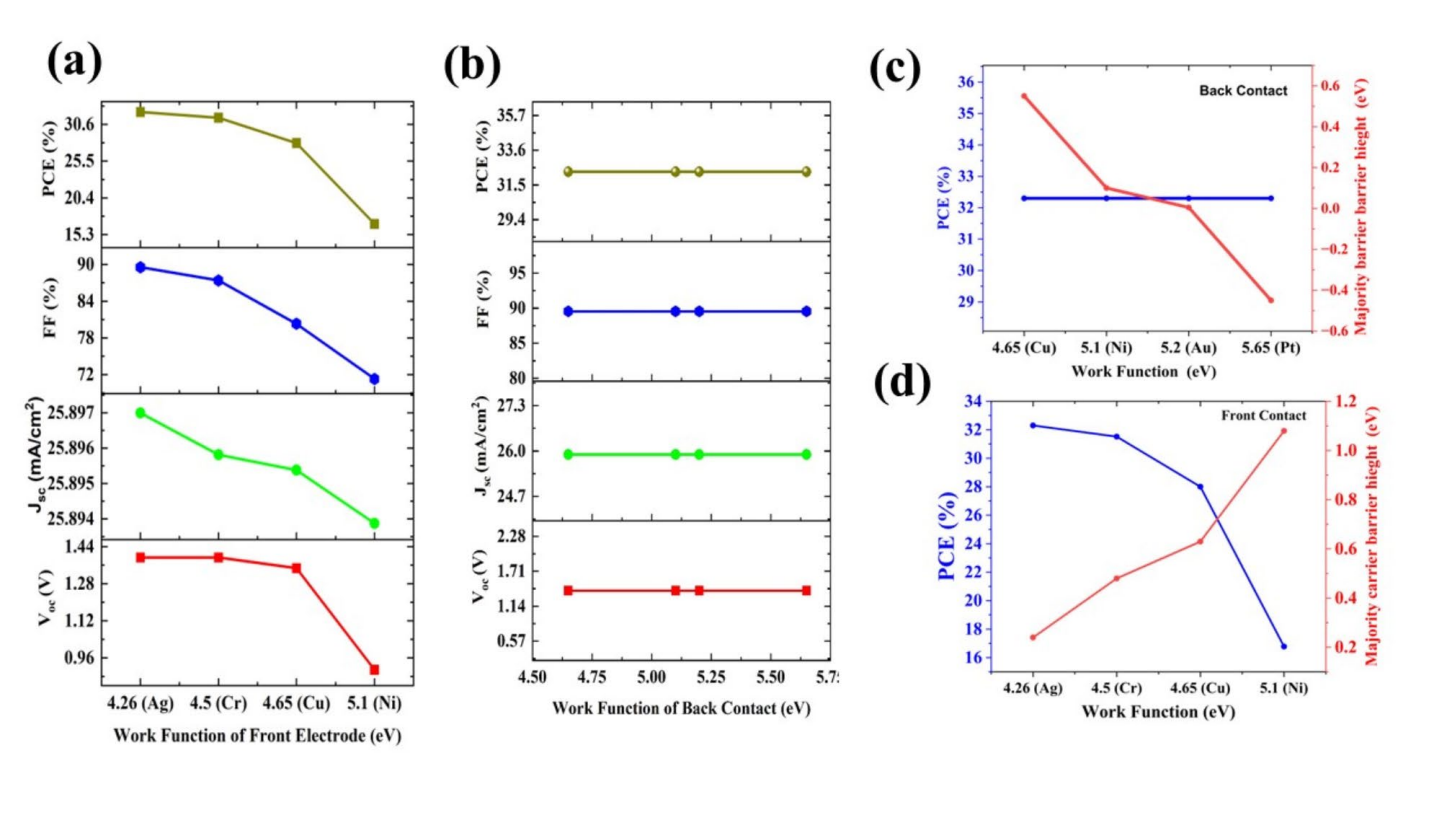
The effect of work function with different metals in the (**a**) front contact and (**b**) back contact on photovoltaic parameters for the configuration Ag/FTO/CuBi_2_O_4_/GQD/Au under ideal conditions. The majority carrier barrier height is denoted (red line) and PCE (blue line) with the different choices of the (**c**) front contact and (**d**) back contact.

The appropriate front and back contact is a crucial element for the extraction of charge carriers. In this case, Ag (4.26 eV), Cr (4.50 eV), Cu (4.65 eV), Ni (5.1 eV), Au (5.2 eV), and Pt (5.65 eV)^43^ are chosen as front and back electrodes (Fig. 5a and b). However, the Au and Pt are not suitable for front contact, and Ag and Cr are not suitable for back contact due to the mismatching with the bands of FTO and HTL, respectively. The variation of work function vs the majority carrier barrier height and PCE are plotted in the Fig. 5c and d. The Figure shows that the decrement in the majority carrier barrier height to the conduction band of FTO reduces the PCE from 32.30% to 16.78%. For smooth conduction of electrons, the appropriate front contact is necessary, and the Ag is fixed as the front contact. In the case of the work function of back contact the PV parameters have little effect due to the saturated conduction of holes from the HTL layers and the Au is kept as back contact by considering the good conductivity of the material. For the champion cell, P V parameters obtained are 1.3928 V (V_oc_), 28.897 mA/cm^2^ (J_sc_), 89.55% (FF), and PCE of 32.30%.

#### Quantum efficiency

**Fig. 6:**
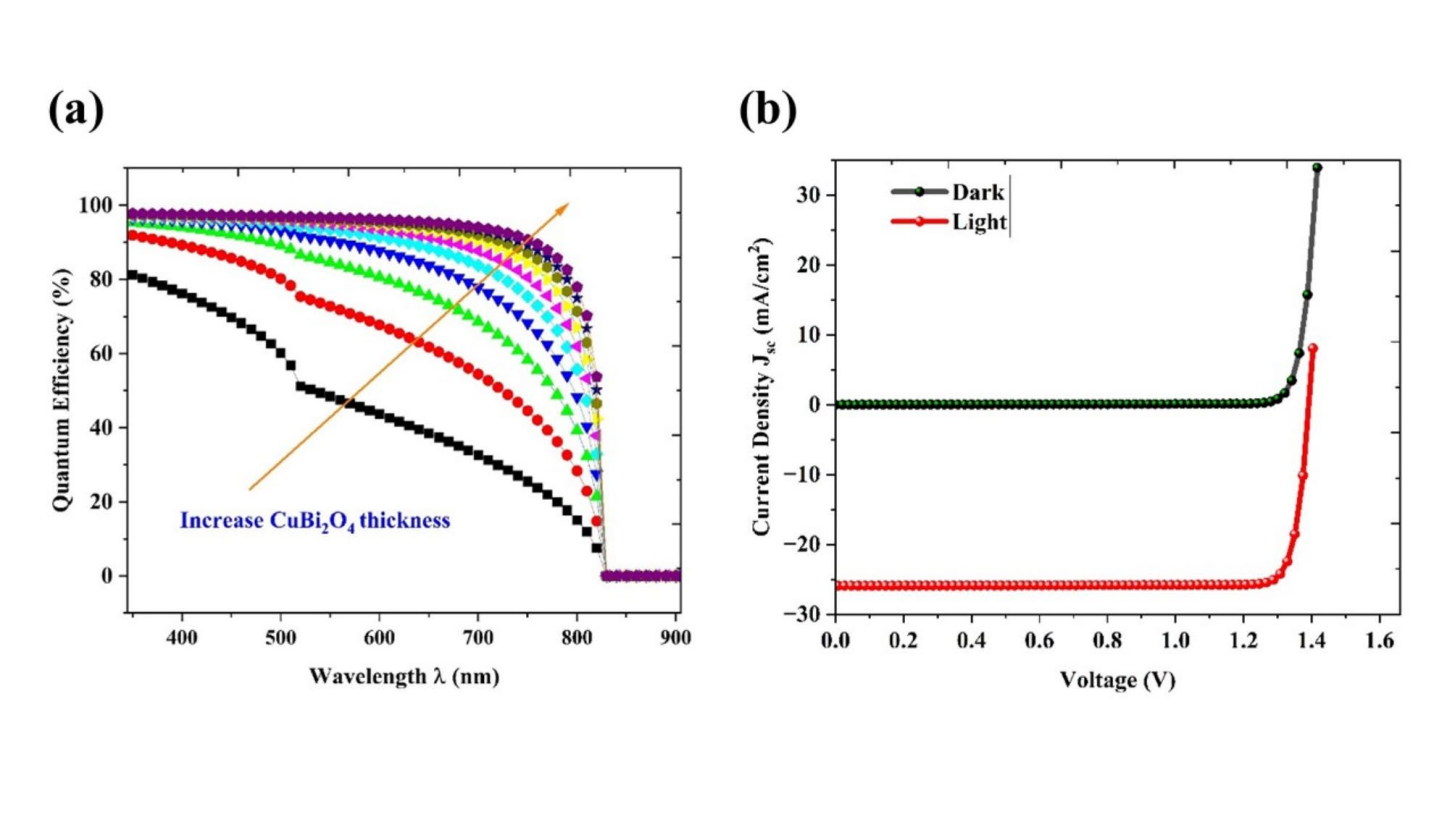
(**a**) Quantum efficiency of the proposed Ag/FTO/CuBi_2_O_4_/GQD/Au cell with a variation of thickness CuBi_2_O_4_ from 100 to 1000 nm (**b**) The J-V characteristics for the optimized devices in dark (green dotted lines) and light (red dotted line) under ideal conditions.

Quantum efficiency (QE) is the conversion of photon energy to exciton generation, which can participate in the current generation. The QE is calculated with variation in the thickness of the absorber layer from 100 to 1000 nm (Fig. 6a). The absorption range spans from 300 to 820 nm which is almost a major portion of the solar spectrum. An increase in thickness leads to an improvement in quantum efficiency (QE) due to a higher number of photons over the entire spectrum. The effectiveness of QE is improving, and the current generation is proportionally increasing.

#### The champion cell performance

The champion cell performance was analyzed in the configuration of Ag/FTO (100* nm*)/CuBi_2_O_4_ (1000 nm) /GQD (100 nm) /Au by J-V characteristics in both dark and bright conditions, which are plotted (Fig. 6b). The J-V characteristic shows the increment in current density in light conditions, the dark condition shows a typical p–n junction behavior in the J-V plot and there will be a negative current in the light condition which shows the current generation. The champion cell achieved an efficiency of 32.30% with V_oc_ of 1.39 V J_sc_ of 25.89 mA/cm^2^ and FF of 89.55%.

### Non-ideal conditions

Under non-ideal conditions, the effect of parasitic resistance, defect density, radiative, and auger recombination losses are analyzed. The additional parameters used for nonideal conditions are listed in Table 3.

**Table 3:** Parameters for nonideal conditions for the configuration Ag/FTO/CuBi_2_O_4_/GQD/Au.

Parameters	CuBi_2_O_4_ (Absorber Layer)	GQD (HTL)
Carrier lifetime (ns)	32^44^	24^45^
Defect density	10^12^*	10^12^*
Radiative Recombination Coefficient (B_r_)	3.125 × 10^–12^*	4.17 × 10^–11^*
Auger Recombination Coefficient (B_Auger_)	3.125 × 10^–31^*	4.17 × 10^–29^*

*Variable parameters

#### Effect of parasitic resistances

**Fig. 7:**
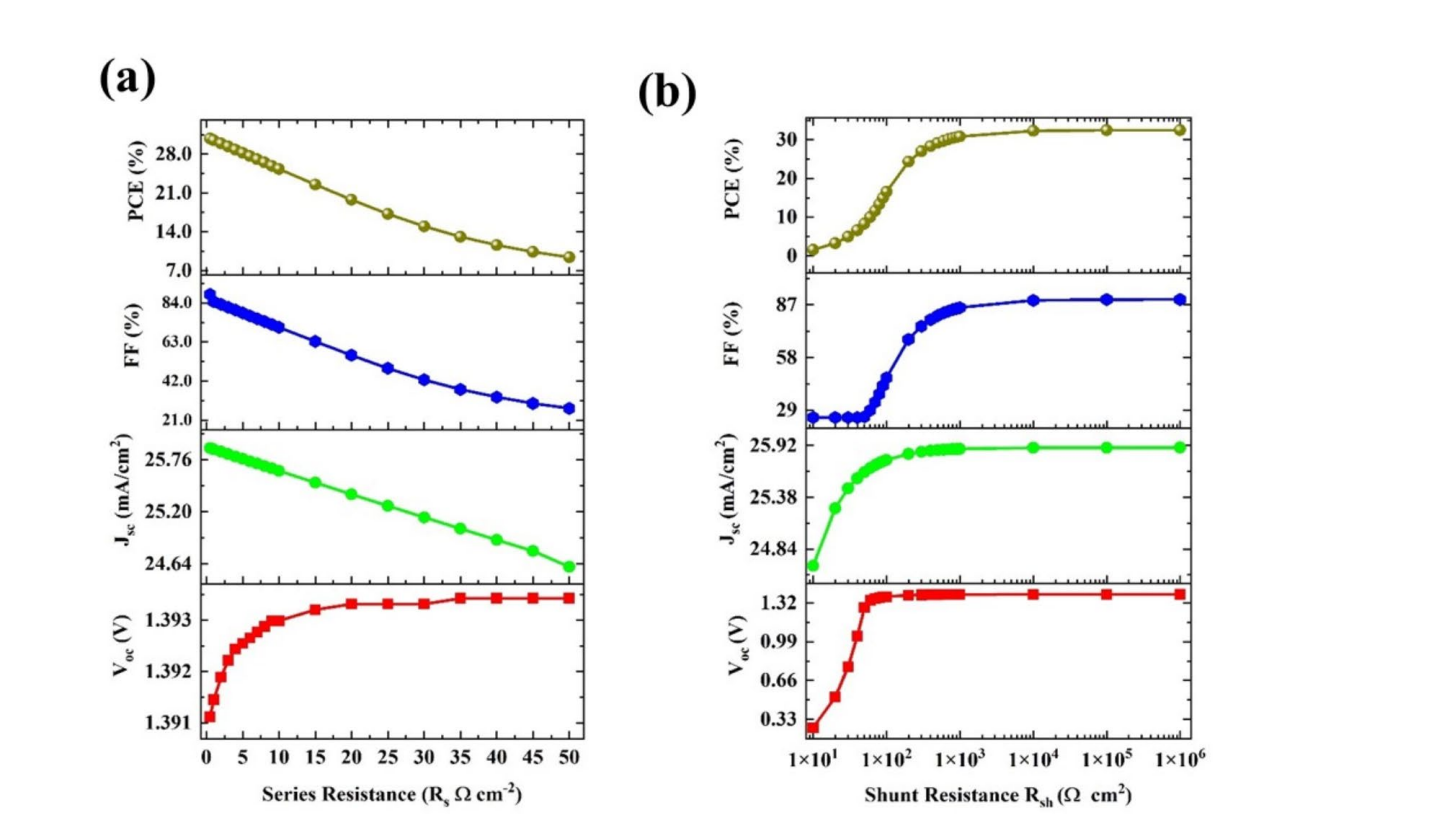
The variation of photovoltaic parameters (V_oc_, J_sc_, FF, and PCE) upon the variation of (**a**) series resistances from 0.5 Ω cm^2^ to 50 Ω cm^2^, and (**b**) shunt resistance from 10 Ω cm^2^ to 10^6^ Ω cm^2^ for the configuration of Ag/FTO/CuBi_2_O_4_/GQD/Au under non-ideal conditions.

**Fig. 8:**
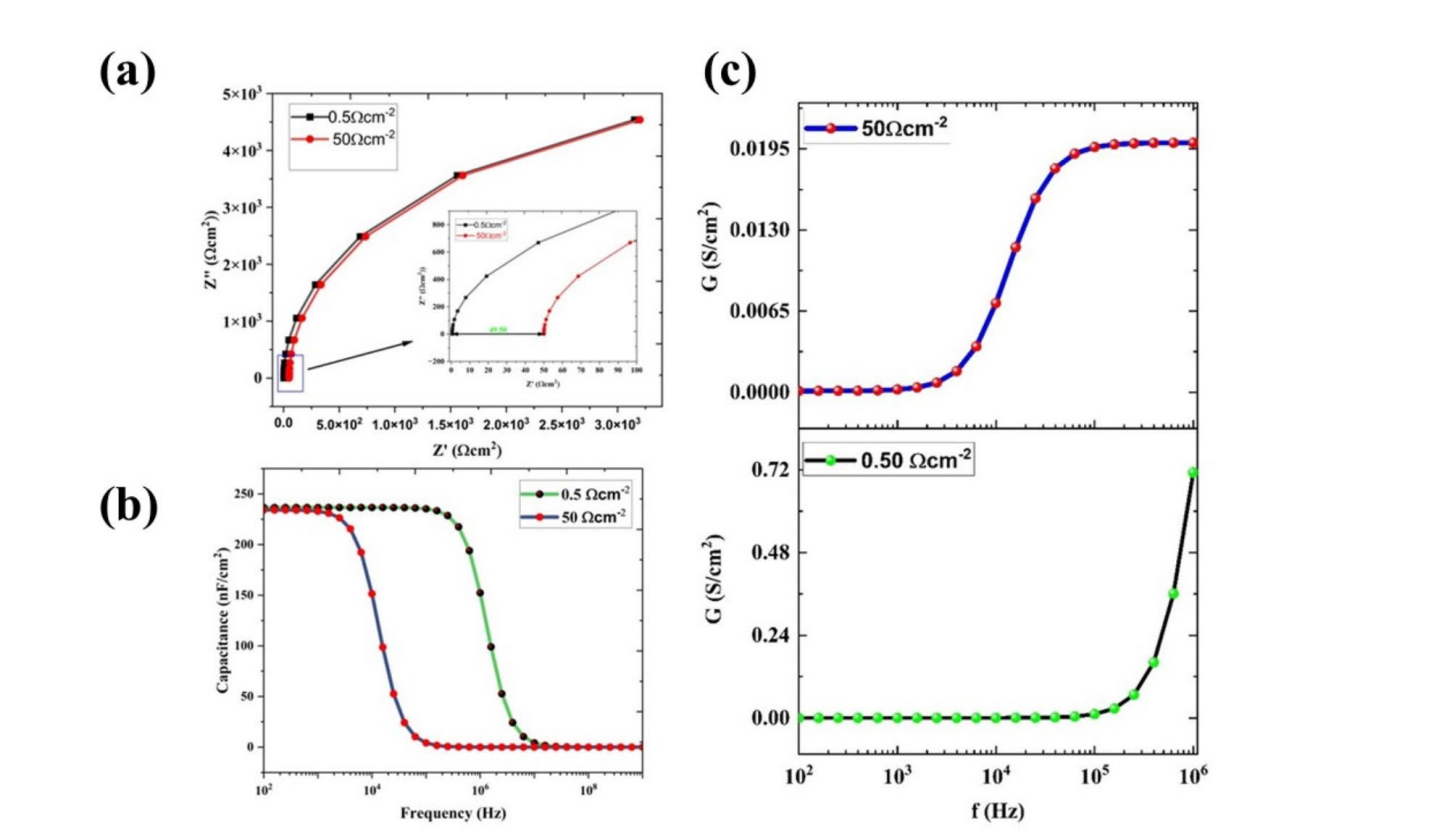
(**a**) Nyquist plot for the comparison of the series resistance at 0.5 Ω cm^2^ and 50 Ω cm^2^ (**b**) Capacitance Vs frequency plot. The conductivity Vs frequency plot for two different shunt resistances (**c**) 50 and (**d**) 0.5 Ω cm^2^ for the configuration of Ag/FTO/CuBi_2_O_4_/GQD/Au cell under non-ideal conditions.

In the ideal condition, we consider the contact between the layers to be negligible resistance. The practical cell has parasitic resistances which will adversely affect the FF and hence the PV parameters. The series resistance is from the cumulative contribution of resistance from the contacts, between layers, and interfaces of metal and semiconductor^46^. In this study, we varied the series resistance from 0.5 Ω cm^2^ to 50 Ω cm^2^, and the PCE was drastically reduced from 30.80 to 9.39% (Fig. 7a). This drastic reduction is mainly due to the fall of FF from 88.501% to 27.38%. The J_sc_ values also reduced from 25.88 to 24.66 mA/cm^2^ may be due to the restriction in the pathways of layers. The analysis of the series resistance has been done using C-V analysis. To quantify the effect of series resistance, the Nyquist plot for the comparison is plotted in two series resistances of 0.5 Ω cm^−2^ and 50 Ω cm^−2^ as shown in Fig. 8a. It is observed that there is a shift in the real impedance of 49.5 Ω cm^−2^ (inset of Fig. 8a). The capacitance vs frequency (C-f) was analyzed upon the increase of series resistance from 0.5 to 50Ω cm^−2^, and the reduction capacitance value shifted from high frequencies to low frequencies. This, confirms that the increment in series resistance will affect the capacitive nature of the device and the charge kinetics. The conductivity and frequency graph indicates that there is a huge reduction in the conductivity due to resistance which will reduce the overall performance of the cells (Fig. 8b,c).

The shunt resistance is due to leakage across the junction, which mainly comes from defects and impurities in the layers. Here, we varied the R_sh_ from 10Ω cm^2^ to 10^6^Ω cm^2^, and the PCE increased from 1.60% to 32.40%. The other PV parameters are also increased such as V_oc_ from 0.26 V to 1.39 V, J_sc_ from 24.66 mA/cm^2^ to 25.89 mA/cm^2^, and FF from 25.0 to 89.9% (Fig. 7b). The fill factor has one of the major impacts from shunt resistance and the voltage is also reduced due to the leakages of charges. In the optimized condition, the series resistance was kept at 0.5Ω cm^2^, and the shunt resistance was at 10^6^ Ω cm^2^, and the extracted PV parameters were 1.39 V (V_oc_), 25.89 mA/cm^2^ (J_sc_), 89.55% (FF), and 32.30 (PCE).

#### The effect of defect densities

**Fig. 9:**
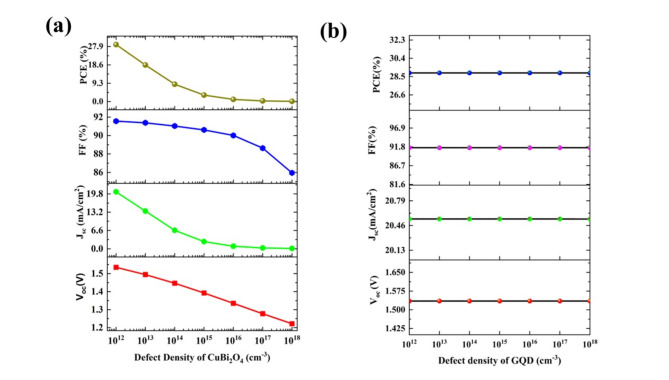
The variation of photovoltaic parameters (V_oc_, J_sc_, FF, and PCE) upon the variation of defect density of (**a**) CuBi_2_O_4_ and (**b**) GQD and for the proposed cell configuration of Ag/FTO/CuBi_2_O_4_/GQD/Au under non-ideal conditions**.**

The defect density is one of the crucial parameters to achieve high performance and the defects mainly arise from the faults in the fabrication methods. The defect density of CuBi_2_O_4_ varied from 10^12^ cm^−3^ to 10^18^ cm^−3^. As the defect density increases, the PCE is abruptly decreasing from 28.88% to 0.12%. This main reduction is due to the current reduction due to the recombination in the defect sites, and the current density is reduced by 99.5% of its original value (from 20.54 mA/cm^2^ to 0.11 mA/cm^2^). The other parameters the V_oc_ (1.53 V to 1.22 V) and FF (91.56 to 85.94%) were also reduced (Fig. 9a). The defect in absorber density would scrunch the cell performance.

The defect density in the HTL layer also varied similarly to the absorber layer (from 10^12^ cm^−3^ to 10^18^ cm^−3^). The PV parameters are almost constant for all defect densities (Fig. 9b) because no electrons are transported to the HTL layer for recombination, and only the hole is transported through the GQD layer. In short, the recombination does not occur in the GQD layer due to the unavailability of electrons. This shows that the GQD material here can act as an electron-blocking layer. The V_oc_, J_sc_, FF, and PCE are 1.53 V, 20.54 mA/cm^2^, 91.56%, and 28.8% respectively. For the champion cell, both HTL and absorber layer defect densities are kept to 10^12^ cm^−3^ ^22^.

#### The effect of radiative and auger recombination

The radiative and Auger recombinations are included further to evaluate the performance of SCs. The coefficients of radiative recombination and Auger recombination are calculated by using the formulae^25^

7 $${B}_{r}=\frac{1}{{\tau }_{n, rad }\,\,or\,\, {\tau }_{p, rad } {N}_{A} or{N}_{D}}$$

8 $${B}_{Auger,n or p}=\frac{1}{{\tau }_{n,Auger }\,\, or\,\, {\tau }_{p,Auger } {{N}_{A}}^{2} or{{N}_{D}}^{2}}$$

The τ_n,rad,_ and τ_p,rad_ are the electron and hole radiative lifetime, and *N*_*A*_, and *N*_*D*_ are acceptor and donor density. The parameters chosen for radiative recombination are given in the Table 4.

**Table 4:** The current components from both ideal and nonideal conditions at V = 0.

Condition	J_generation_ (mA/cm^2^)	J_SRH_ (mA/cm^2^)	J_Auger_ (mA/cm^2^)	J_rad_ (mA/cm^2^)	J_recomb_ (mA/cm^2^ )	J_total_ or J_sc_ (mA/cm^2^)
Ideal	33.50	7.54	0.00	0.00	7.60	25.89
Non-ideal	33.50	0.02	16.02	16.02	32.06	1.44

**Fig. 10:**
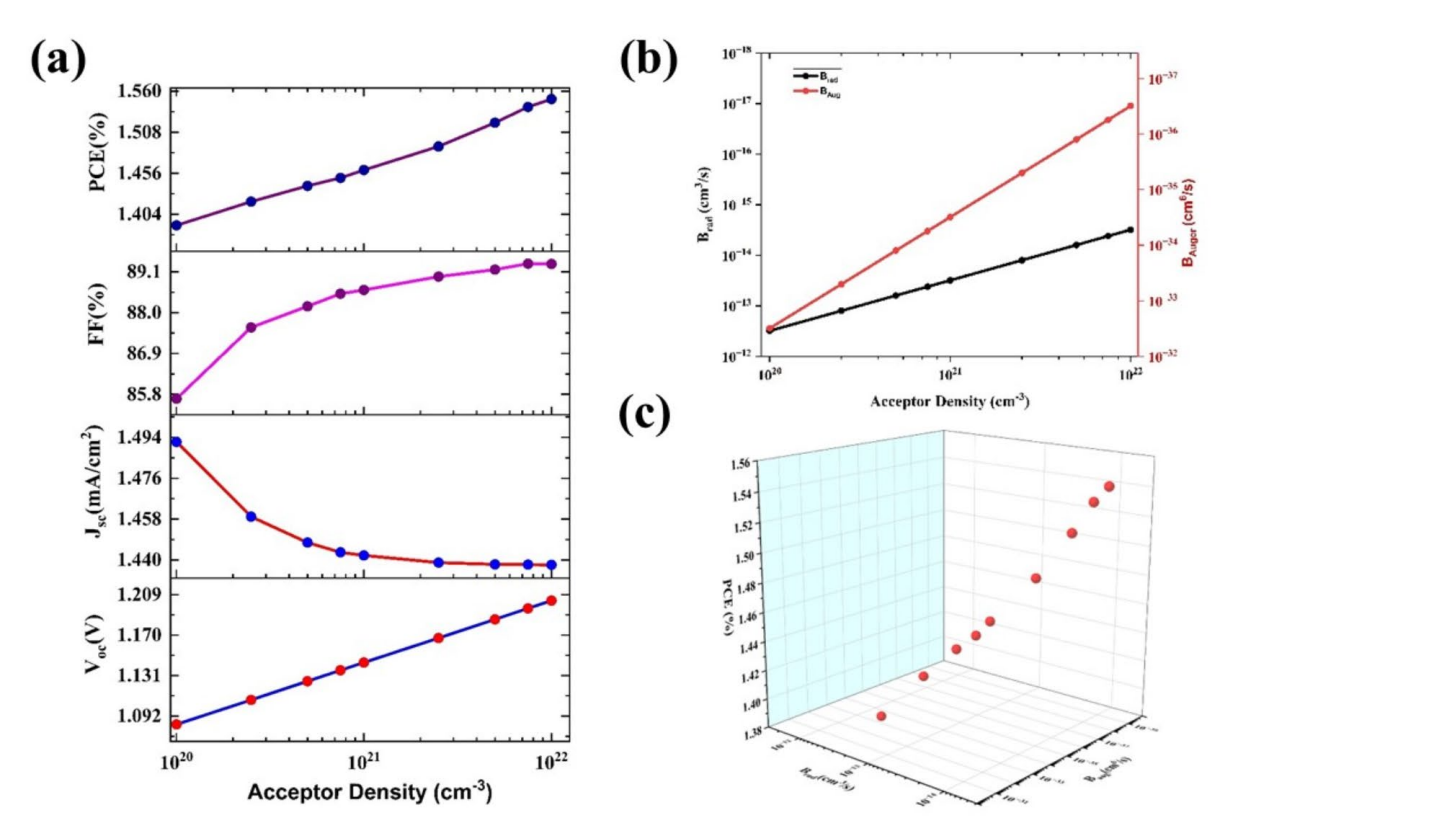
(**a**) The variation of photovoltaic parameters (V_oc_, J_sc_, FF, and PCE) upon the variation of acceptor density of absorber layer of the proposed cell configurations of Ag/FTO/CuBi_2_O_4_/GQD/Au under non-ideal conditions (**b**) Plot between acceptor density of absorber layer and recombination coefficients (**c**) 3D plot for the PCE variation with respect to the recombination coefficients.

The carrier lifetime for CuBi_2_O_4_ reported from the experimental data is 32 ns^44^ and the acceptor density varied from 10^18^ to 10^22^ cm^−3^. The variation of both coefficients with the acceptor density is given in the Fig. 10b. This introduction of radiative and Auger recombinations drastically reduced the PCE from 28.80% to 1.55%. However, it is observed that the improvement of PCE from 1.39% to 1.55% upon the increase of acceptor density (from 10^18^ cm^−3^ to 10^22^ cm^−3^) (Fig. 10a). This is due to a slight reduction in both recombination coefficients upon the increase of acceptor density, which further reduces the recombination (Fig. 10b,c). The other PV parameters are increased such as V_oc_ from 1.08 V to 1.20 V, FF 85.68 to 89.31% while there is a decrement in J_sc_ 1.49 to 1.43 mA/cm^2^. The increase in carrier lifetime may reduce the recombination factor and there are more experimental studies needed to alter the optoelectronic properties of CuBi_2_O_4_.

##### I–V characteristics

**Fig. 11:**
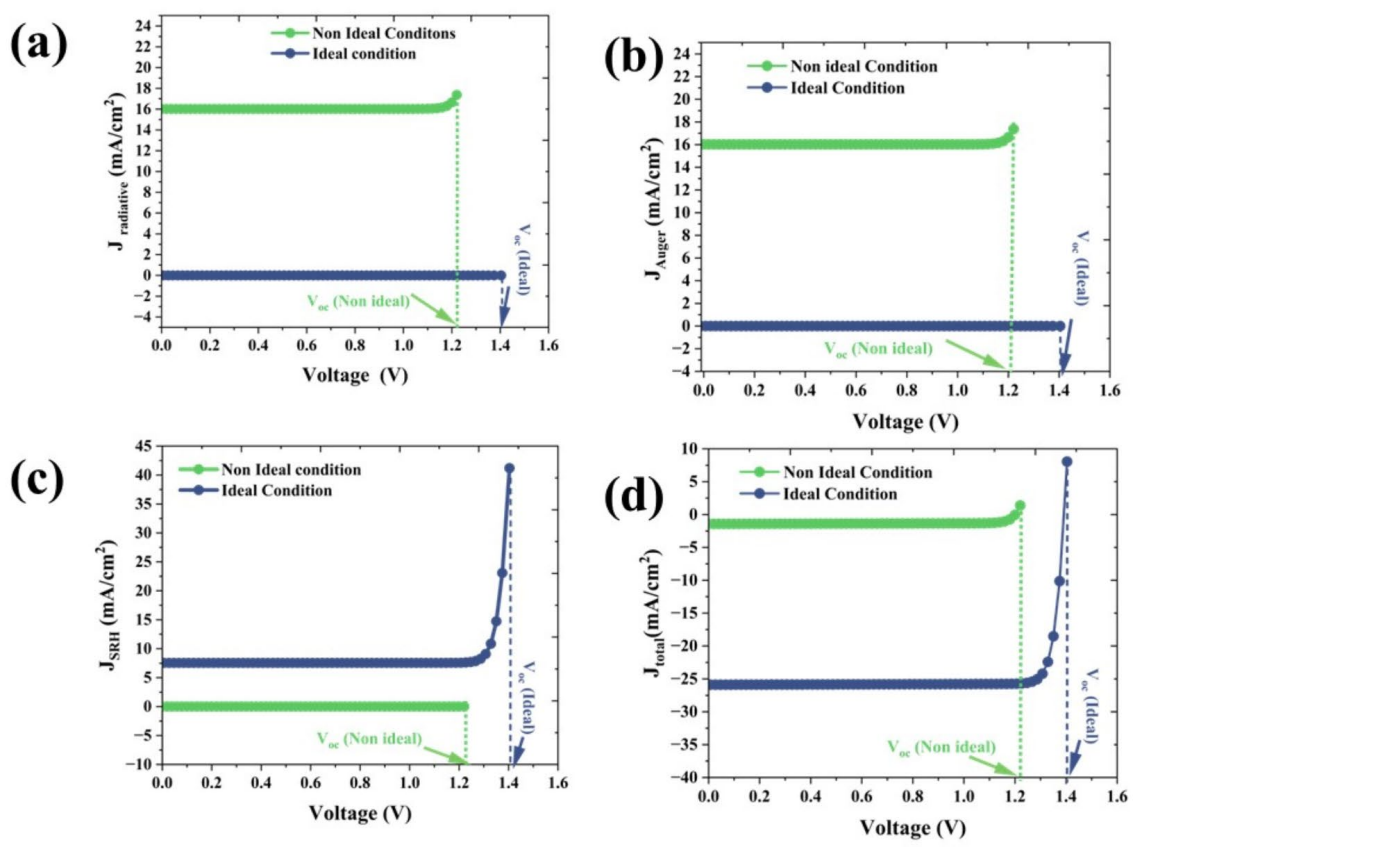
The contributions of current densities of ideal (blue line) and non-ideal (green line) conditions (**a**) J_radiative_ (**b**) J_auger_ (**c**) J_SRH_ and (**d**) J_total_. The vertical dotted lines represent the open circuit voltage for the ideal condition (blue dotted lines) and non-ideal conditions (green dotted line) kept as the cut-off voltage for analysis.

The current–voltage characteristics are studied for ideal and non-ideal conditions shown in Fig. 11. The generation of current (J_generation_), Shockley–Read–Hall (SRH) recombination (J_SRH_) current, radiative (J_rad_), and Auger (J_Auger_) are calculated for ideal and non-ideal conditions and the results are presented in Table. Considering the generation of current (J_generation_), in both cases the values are the same value, 32.50 mA/cm^2^ which reveals, that the non-ideal conditions of the generation of current are the same. The Auger and radiative factors are almost zero in the case of ideal conditions, and the non-ideal conditions both are about 16 mA/cm^2^ (Figs. 11a,b) which makes the J_sc_ of 1.44 mA/cm^2^_._ The J_SRH_, values are 7.54 mA/cm^2^ and 0.02 mA/cm^2^ for the case of ideal and nonideal conditions, respectively (Fig. 11c,d). The reduction in J_SRH_ in nonideal conditions may be due to the dominance of the other recombination.

#### The reflection losses

**Fig. 12:**
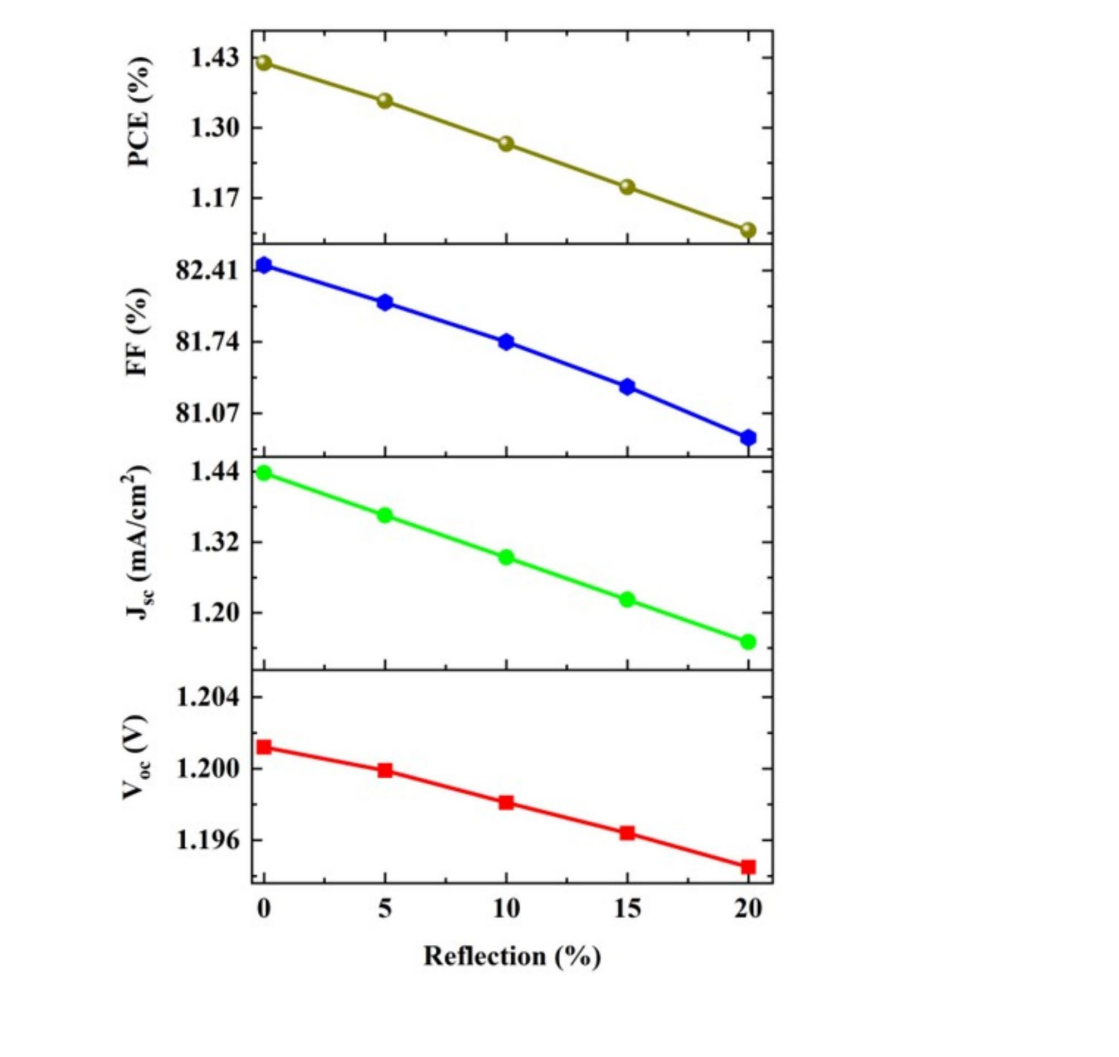
The variation of photovoltaic parameters (V_oc_, J_sc_, FF, and PCE) upon the varying reflection loss of the proposed cell structure Ag/FTO/CuBi_2_O_4_/GQD/Au under non-ideal conditions.

In real conditions, the transparency of the substrate is maintained to be maximum to get better efficiency. There will be a reduction of about 10–15% loss of light from the layer due to reflection losses^47^. The reflection parameter is varied from 0 to 20% loss (Fig. 12) and these reflection losses lead to a decrease in all PV parameters due to less absorption of light and a reduction of exciton generation. This further leads to the reduction of PCE from 1.35% to 1.11% (18%). The other parameters are reduced such as V_oc_ (from 1.2 to 1.19 V), J_sc_ (1.43 mA/cm^2^ to 1.15 mA/cm^2^), and FF from 82.46% to 80.84%. However, the reflection losses can be reduced by using anti-coating reflection coatings and surface structuring.

### Thickness effect comparison of ideal and non-ideal conditions

#### Effect of layer thickness

**Fig. 13:**
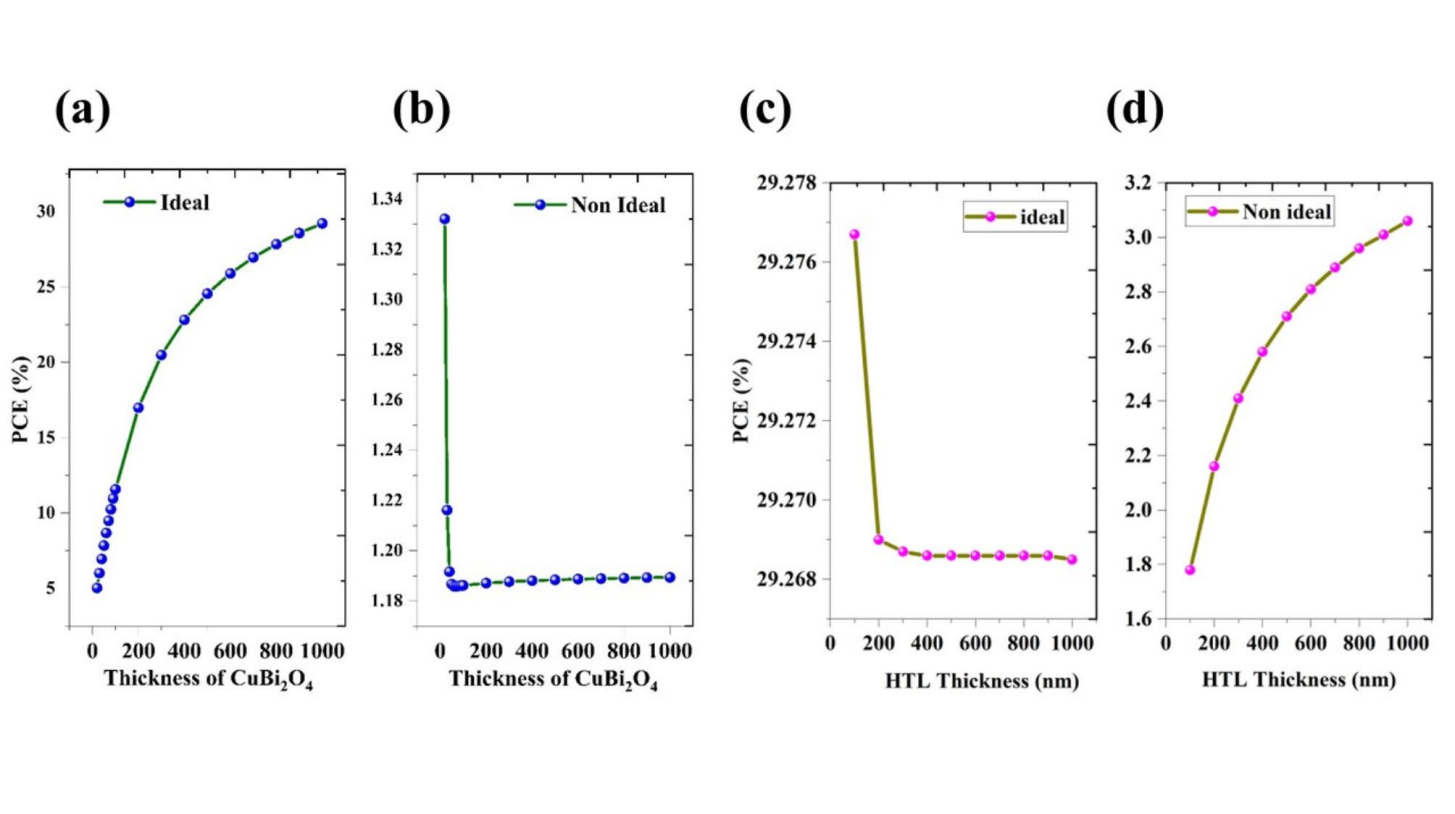
The variation of PCE upon the variation of thickness of CuBi_2_O_4_ under (**a**) ideal (**b**) nonideal conditions and thickness of HTL under (**c**) ideal (**d**) nonideal conditions of the proposed cell configurations of Ag/FTO/CuBi_2_O_4_/GQD/Au.

**Fig. 14:**
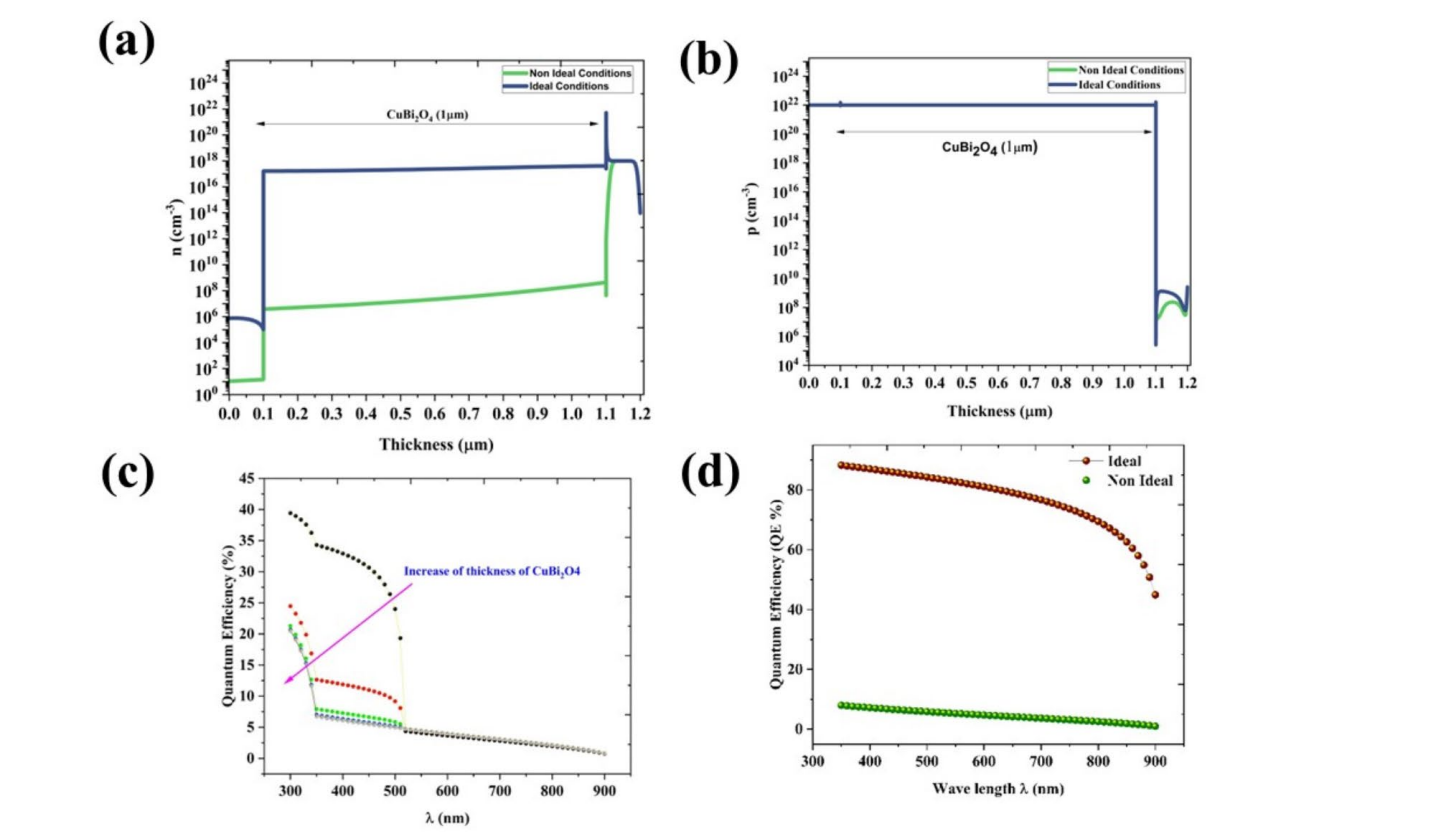
(**a**) electron and (**b**) hole concentration of the champion cell where HTL thickness of 100 nm and absorber layer thickness of 1 μm, in ideal (blue line) non-ideal (green line) conditions. (**c**) QE for various thicknesses of copper bismuth oxide in non-ideal conditions and (**d**) comparison of QE with ideal (yellow line) and non-ideal conditions (green line) for an absorber layer thickness at 1 μm.

The comparison of the effect of thickness was done by the variation of both the absorber layer and HTL (from 10 to 1000 nm) in ideal and nonideal conditions. In an ideal, the PV parameters are improved by the variation of the absorber layer (Fig. 13a). Interestingly, in the non-ideal conditions, the PCE is reduced from 1.77 to 1.18% by the varying thickness from 10 to 1000 nm and this is due to heavy recombination and small carrier diffusion length (Fig. 13b). The carrier diffusion length can be obtained by the Eq. (9)^48^

9 $${L}_{n}=\sqrt{{D}_{n} \tau }$$

where, *D*_*n*_ is the diffusion coefficient ${D}_{n}=\frac{{\mu }_{n}kT}{e}$, *μ*_*n*_ is the mobility of electrons, *k* is the Boltzmann constant, *T* is temperature, and *e* is the charge of electrons. The calculated value of the diffusion length of CuBi_2_O_4_ is around 13 nm. The thickness of the HTL layer has little effect on PCE in the ideal conditions (Fig. 13c), while the PCE has an enhanced from 1.8% to 3.09% in nonideal conditions (Fig. 13d) which shows that the GQDs can extract more holes and reduce the recombination. The electron and hole density plot (Fig. 14a,b) shows that hole concentration is almost the same for both ideal and non-ideal conditions while there is a large difference in the electron density due to the recombination. The QE plot for the non-ideal condition reveals that the QE is very low compared with the ideal condition and the values decline for the increase of absorber layer thickness (Fig. 14c). Further, 95% decline in QE in nonideal conditions which is consistent with the result of PCE (from 33.59% to 1.42%). The major current loss is due to the recombination factor, which suppresses the photon excitation generation depicted in the QE calculation (Fig. 14d).

##### Summary of simulation

The summary of the simulation with each step is depicted in Table 5.

**Table 5:** Summary of simulation of the proposed Ag/FTO/CuBi_2_O_4_/GQD/Au cell structure from ideal to non-ideal conditions.

Steps	Conditions	V_oc_ (V)	J_sc_ (mA/cm^2^)	FF (%)	PCE (%)
1	Absorber layer thickness (1 μm)	1.4227	23.19719	88.53	29.22
2	Absorber layer Acceptor density 10^22^ cm^−3^	1.5555	22.85834	91.64	32.59
3	The band gap of the absorber layer (1.35 eV)	1.3926	25.89853	90.92	32.79
4	HTL Acceptor density 10^22^ cm^−3^	1.5615	23.69637	91.62	33.9
5	The shunt resistance 10^4^ Ω cm^−2^	1.3928	25.89719	89.55	32.3
6	The series resistance 0.5 Ω cm^−2^	1.3915	25.88554	88.5	30.8
7	Applying the defect density of the absorber layer	1.535	20.54709	91.56	28.88
8	Adding the radiative recombination coefficient	1.2192	1.98341	84.47	2.04
9	Adding Auger coefficient	1.2012	1.43777	82.46	1.42
10	Adding a 15% reflection of losses	1.1964	1.22211	81.32	1.19
11	GQD thickness 1000 nm	1.2233	2.90424	86.13	3.06

## Conclusions

The simulation studies reveal that the solar cell structure of Ag/FTO/CuBi_2_O_4_/GQD/Au has higher efficiency in ideal conditions which is about 33.9%. Here, GQDs can act as not only the HTL layer but it can also as an electron-blocking layer. Firstly, fixing the thickness of the absorber layer at 1000 nm, the PV parameters such as 1.42 V (V_oc_), 23.19 mA/cm^2^ (J_sc_), 88.53% (FF), and PCE of 29.22% are obtained. In the next step, the increment of acceptor density to 10^22^ cm^−3^, increased the PCE to 32.59% mainly due to the enhancement of built-in potential. Further, the tuning of the band gap enhanced the PCE to 32.59% for a band gap of 1.35 eV which is mainly due to the increment in current from 22.58 mA/cm^2^ to 25.89 mA/cm^2^. The PCE again raised to 33.9% by HTL acceptor density is set to 10^22^ cm^−3^_._ The introduction of parasitic resistance reduces the PCE to 32.3%, 30.8% for a shunt resistance (10^6^ Ω cm^−2^), and series resistance of 0.5 Ω cm^−2^, respectively. Applying a defect density of 10^12^ to both absorbers and HTL leads to the PCE of 30.8% to 28.88%. Considering the recombination factors of radiative and Auger recombination, the PCE is down falling to 2.04 and 1.42%, respectively. This reduction is mainly from the reduction in current density from 20.45 mA/cm^2^ to 1.21 mA/cm^2^. The reflection losses further decrease the PCE from 1.42% to 1.19% in the case of 15% reflection losses. However, enhancement of PCE from 1.19% to 3.06% was observed with the HTL thickness of 1000 nm which arises from the efficient charge extraction.

## Data Availability

All data generated or analyzed during this study are included in this article.
